# A Soft Polydimethylsiloxane Liquid Metal Interdigitated Capacitor Sensor and Its Integration in a Flexible Hybrid System for On-Body Respiratory Sensing

**DOI:** 10.3390/ma12091458

**Published:** 2019-05-06

**Authors:** Yida Li, Suryakanta Nayak, Yuxuan Luo, Yijie Liu, Hari Krishna Salila Vijayalal Mohan, Jieming Pan, Zhuangjian Liu, Chun Huat Heng, Aaron Voon-Yew Thean

**Affiliations:** 1 Department of Electrical and Computer Engineering, National University of Singapore, 4 Engineering Drive 3, Singapore 117583, Singapore; suryakanta.nayak@nus.edu.sg (S.N.); elelyux@nus.edu.sg (Y.L.); liuyijie1987@outlook.com (Y.L.); harikrishnasv@nus.edu.sg (H.K.S.V.M.); e0361620@u.nus.edu (J.P.); elehch@nus.edu.sg (C.H.H.); 2 Institute of High Performance Computing, A*STAR Research Entities, 1 Fusionopolis Way, #16-16 Connexis, Singapore 138632, Singapore; liuzj@ihpc.a-star.edu.sg

**Keywords:** stretchable, polydimethylsiloxane, liquid-metal, capacitor

## Abstract

We report on the dual mechanical and proximity sensing effect of soft-matter interdigitated (IDE) capacitor sensors, together with its modelling using finite element (FE) simulation to elucidate the sensing mechanism. The IDE capacitor is based on liquid-phase GaInSn alloy (Galinstan) embedded in a polydimethylsiloxane (PDMS) microfludics channel. The use of liquid-metal as a material for soft sensors allows theoretically infinite deformation without breaking electrical connections. The capacitance sensing is a result of E-field line disturbances from electrode deformation (mechanical effect), as well as floating electrodes in the form of human skin (proximity effect). Using the proximity effect, we show that spatial detection as large as 28 cm can be achieved. As a demonstration of a hybrid electronic system, we show that by integrating the IDE capacitors with a capacitance sensing chip, respiration rate due to a human’s chest motion can be captured, showing potential in its implementation for wearable health-monitoring.

## 1. Introduction

Soft electronics that enable conformal contacts on irregular surfaces is an emerging area with increasing importance. The development of this technology is expected to enhance human–machine interfaces to cover areas such as medical and e-health applications, robotics, and communications [1,2,3,4,5,6,7,8,9,10,11,12]. Flexible and stretchable electronics are vigorously studied for the choice of materials and integration strategies [5,6,7,9,10,13]. However, flexible electronics is only suitable for a non-conformal substrate that is static or does not undergo significant strain in the x-y axes during operation [7,13]. Stretchable electronics, on the other hand, offers more degrees of freedom that can theoretically tolerate mechanical strain in all three axes. This leads to increasing interest in the field of wearable devices for health-monitoring. In such applications, soft sensors with a Young’s modulus that matches that of skin are particularly attractive. This allows for prolonged human body attachment for continuous health monitoring [5,9,13,14]. Hence, besides the functionality of the sensor, there is a need to address the mechanical robustness and the system integration strategies with high performance integrated circuits (IC) [5,7,9,13,15,16,17,18]. This calls for novel materials and approaches, such as the use of liquid metal or stretchable conducting materials, in place of conventional metal materials that are rigid [19,20,21]. Several studies have reported soft-matter capacitors comprising of a microfludics channel of liquid-metal (Galinstan) and described the theoretical model of capacitance change effect under mechanical deformation [22,23]. While there are reports of sensors with similar structures being used for bio-sensing and proximity sensing, the implementation of such sensors in real systems is still lacking [24,25].

In this work, we report on the experimental characterization together with a finite element (FE) simulation model of soft interdigitated (IDE) capacitors sensors. The IDE capacitor was fabricated from serpentine microfluidics channels of GaInSn liquid alloy (Galinstan) embedded in a polydimethylsiloxane (PDMS) matrix. The soft IDE capacitor, besides responding to perpendicular strains in the x-y axes, also responded to proximity sensing of a human finger that has not been reported before. In addition, previous reports have not described the IDE capacitor using FE simulation with proper boundary conditions. We will address these in this paper. Experimentally, the opposite capacitance change in the x and y directions allows for detection of direction specific strain with a resolution of 0.02 pF/(% engineering strain) as verified by our FE simulation model. Proximity sensing is achieved via the modifications of the capacitor’s fringing E-field by the surrounding dielectric medium [26]. We show that spatial detection as large as 28 cm can be achieved by varying a human’s finger distance to the IDE capacitor. The described FE simulation model provides a design guideline for future implementation of such a class of sensors. Finally, as a demonstration of a hybrid electronic system, we show that when the IDE capacitor sensor attached to a human chest is integrated with a functional capacitance sensing chip, it can be used for on-body respiratory sensing by utilizing the proximity effect, thus demonstrating its potential as a soft sensor suitable for use in wearables for health monitoring.

## 2. Fabrication Process of Soft PDMS-Liquid Metal IDE Capacitor

**Figure 1:**
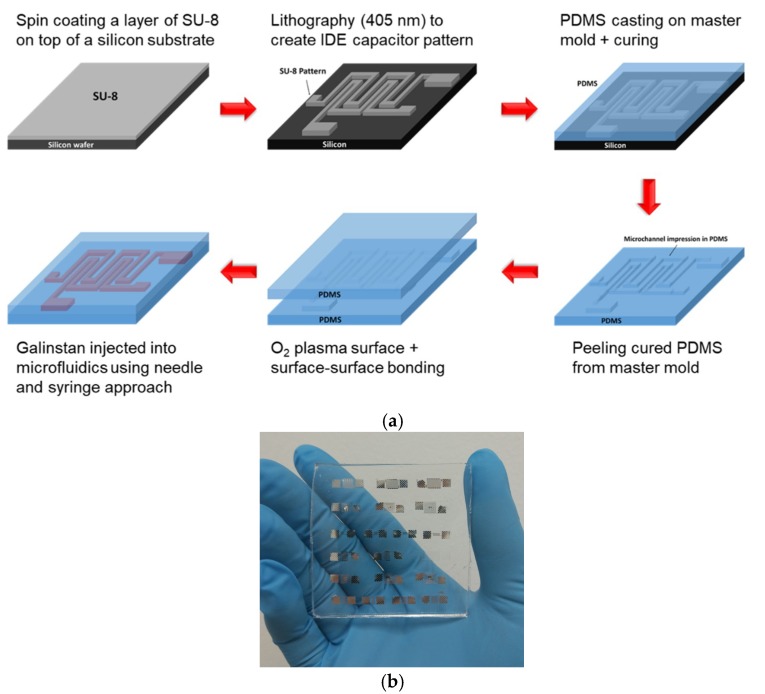
(**a**) Fabrication process flow of the soft IDE capacitor, and (**b**) photo image of the fabricated IDE soft capacitors of various sizes. The height of the liquid metal electrode was 100 µm.

The stretchable capacitor was composed of two layers of PDMS (Dow Corning Sylgard 184). The first layer consisted of a microfluidics channel in the form of an IDE design, fabricated using a soft lithography approach [9]. Special design care in providing for the inlet and outlet points were necessary to ensure the Galinstan filled the microfludics channel properly without trapped air. The master mold was made using a permanent resist (SU-8 3050, Microchem, Westborough, MA, USA), where the PDMS pre-polymer was casted over the pre-fabricated design to a thickness of ~500 µm and removed after curing by peeling. The second layer consisted of a plain piece of PDMS molded to the same thickness of ~500 µm. For the formation of the closed microfluidics channel, the two PDMS layers were surface treated using a light remote O_2_ plasma treatment before contacting with each other. A post-baking step (90 °C, 2 h) ensured a permanent bond of the two layers of PDMS. Finally, the microfluidics channel was completely filled with Galinstan using a needle and syringe approach. The Galinstan was injected from the inlet point while air was extracted from the outlet point simultaneously to allow the complete fill of the microfluidics channel. The height of the Galinstan electrodes was dependent on the master mold, and was 100 µm in this case [9,27,28]. Figure 1a,b shows the fabrication process flow of the soft IDE capacitor, and a photo image of the fabricated IDE soft capacitors with various sizes, respectively.

While encapsulated in the elastomer matrix, the PDMS provided good barrier resistance to moisture as reported earlier, which otherwise would cause the oxidation of the Galinstan [9,28]. We immersed the soft IDE capacitor in water for a period of 10 min and no visible and electrical change to the capacitor were observed. Hence, this allows for its use without degradation in practical environment. For electrical contact to the IDE capacitor, short sections of tungsten (W) wires (Goodfellow, 125 µm, Huntingdon, UK) perforated the PDMS into the Galinstan reservoir and was resealed with PDMS to avoid leakage.

## 3. Electrical Characterization and Modelling of a Static IDE Capacitor

**Figure 2:**
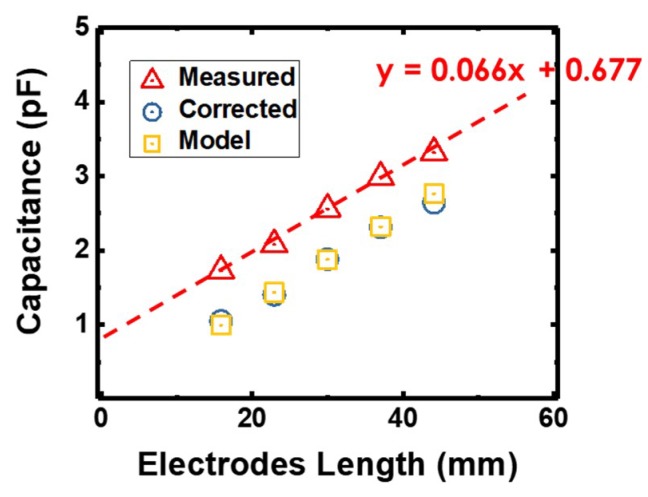
As-measured capacitance across a total electrode length from 15 mm to 44 mm, together with the corrected capacitance values and modeled values based on empirical equations indicated in the legends.

The capacitance of all the fabricated IDE capacitors were first measured and calibrated using a benchtop LCR meter (Keysight E4980A, Santa Rosa, CA, USA) at 1 kHz. In this set of measurements, parasitic capacitance from the connecting wires to the LCR meter caused an offset to the intrinsic capacitance by a fixed positive value. From the linear fit of the data points, the parasitic capacitance was extracted as the y-intercept when the electrode length was zero. Correcting for the parasitic capacitance, the actual capacitance of the IDE capacitors ranged from 1.05 pF to 2.40 pF. Figure 2 shows the as-measured capacitance across a total electrode length from 15 mm to 44 mm, together with the corrected capacitance values and modeled values based on empirical equations. The corrected capacitances agree very well with the capacitor model. It should be noted that the main discrepancy between actual values and measured values comes from the wires’ parasitic capacitances of ~0.68 pF. In subsequent measurements, the wires’ parasitic capacitances were removed from the measured values.

**Figure 3:**
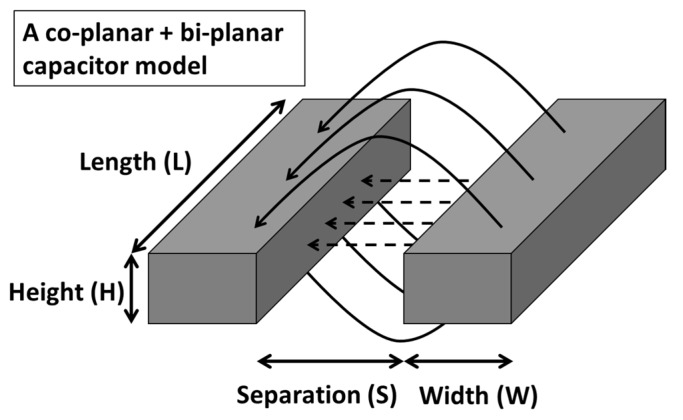
Schematic showing the electric field lines connecting two electrodes of the IDE capacitor. Due to the non-negligible thickness of the electrode, the two contributions of the electric field lines are the direct field lines, and the fringing field lines connected in parallel.

In our IDE capacitor design, the electrodes were of non-negligible thickness, and that was advantageous to enhance its capacitance and sensitivity. As such, a single coplanar capacitor model was not sufficient. Instead, we used a combination of co-planar capacitor and bi-planar capacitor model connected in parallel, as shown by the schematic in Figure 3.

In this model, the electric field lines consist of those that flows directly between the sides of the two electrodes, as well as the fringing field lines that flow from the top and bottom of the electrodes. Further, the effective capacitance of such a structure is described using Equations (1) and (2) for the coplanar model, and Equation (3) for the parallel plate model.

(1) $$C=\;\frac{\epsilon_{r}l\ln\left(-\frac{2}{\sqrt[{4}]{1-\frac{s^{2}}{{\left(s+2w\right)}^{2}}}-1}\left(\sqrt[{4}]{1-\frac{s^{2}}{{\left(s+2w\right)}^{2}}}-1\right)\right)}{377\pi V_{o}},\;\;\;\;\;for\;0<\frac{s}{s+2w}\leq \frac{1}{\sqrt{2}}$$

(2) $$C=\;\frac{\epsilon_{r}l}{377\pi V_{o}\ln\left(-\frac{2}{\sqrt{\frac{s}{s+2w}}-1}\left(\sqrt{\frac{s}{s+2w}}-1\right)\right)},\;\;\;\;\;for\;\frac{1}{\sqrt{2}}<\frac{s}{s+2w}\leq 1$$

(3) $$C=\;\varepsilon_{r}\frac{A}{d}$$

where *ε* is the dielectric constant of the PDMS matrix, *A* is the area of the electrode given by channel length × height of the microfluidics channel, and *d* is the spacing (100 µm) between the electrodes. The above model for a static IDE capacitor was found to be in excellent agreement with experimental values, plotted out together with the measured capacitance in Figure 2, validating the accuracy of the model.

Fassler and co-workers have attempted to study the relationship between the deformation of IDE capacitors and the effect on its capacitance using empirical equations [23]. In their model, they assume that the electrode thickness plays a negligible role in the capacitance, and use only a single co-planar model. However, the thickness of the electrode does contribute to the effective capacitance and should be accounted for as described in Equation (3). In addition, the study is only limited to mechanical strains in the x-y axes. Although model has been reported to predict the change in such a capacitor due to the deformation of the system, it is done by setting assumptions, and does not allow for dynamic boundaries conditions. In addition, the model does not take into account the modifications of electric field lines due to an external change in dielectric material, which we termed as the proximity effect in this work. This effect is similar to reported works on electric-field sensing but has not been discussed thus far for IDE capacitors [26]. Hence, in this work, we further the study by modelling both the IDE capacitor’s deformation and proximity effect using FE simulation. This allows for dynamic boundary conditions to be set. By coupling the simulated results together with experimental measurements, we elucidated the mechanism of capacitance sensing. The FE simulation was performed using Abaqus and the setup is described in Appendix of this paper. Separately, for the following functional tests described in the following sections, a larger IDE capacitor was fabricated in order to achieve a higher sensitivity and easier handling. The new IDE capacitor was fabricated using the same process flow described earlier and the base capacitance was ≈9.6 pF after the parasitic capacitance correction.

### 3.1. Functional Test—Strain Effect

**Figure 4:**
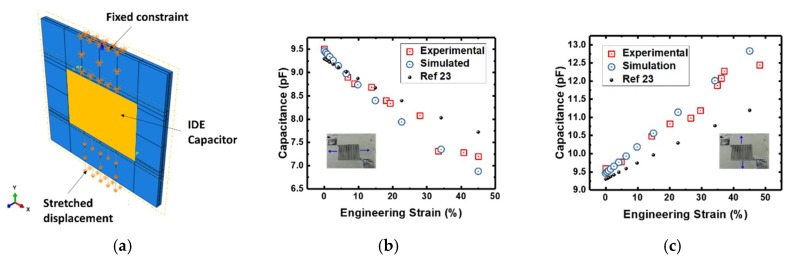
(**a**) FE simulation model where a fixed constraint was applied to one side and a stretched displacement condition was applied to the other side. Stretching in both x- and y-axes was simulated. (**b**) Relationship between capacitance and the x-axis strain. and (**c**) relationship between capacitance and y-axis strain comparison between experimental, FE simulated, and the earlier model proposed [23].

The mechanical deformation of the IDE capacitor led to a change in its geometry and a resultant change in capacitance. As the IDE capacitor was strained in the x-axis (space between electrodes pulled apart), the adjacent electrodes increased in distance from each other, causing a reduction in the capacitance. On the other hand, when the IDE capacitor was strained in the y-axis (electrodes elongating), the effect of the electrode elongating and a decrease in distance between adjacent electrodes resulted in an increase of the capacitance. In the FE simulation, we applied the same boundary condition at both ends of the PDMS during the stretching action. Figure 4a shows the FE simulation model where we applied a fixed constraint on one side and a stretched displacement condition on the other side, while Figure 4b,c shows the experimental measurements, FE simulated values of the capacitance change w.r.t to the two different strain directions up to 50%, and a comparison to the model described by Fassler et al. [23]. Good agreement between experimental and simulated values was obtained, validating the accuracy of the FE model. On the other hand, though showing a similar trend, there existed some error between the measured values as compared to earlier proposed model, thus showing the advantage of FE simulation in such a study [23]. Finally, the IDE capacitor demonstrated a resolution up to 0.02 pF/(% engineering strain).

### 3.2. Proximity Effect

**Figure 5:**
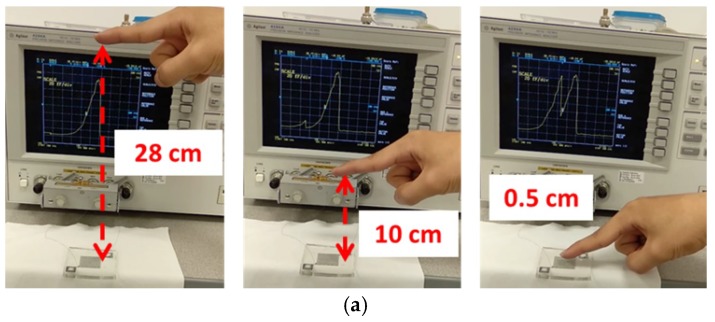

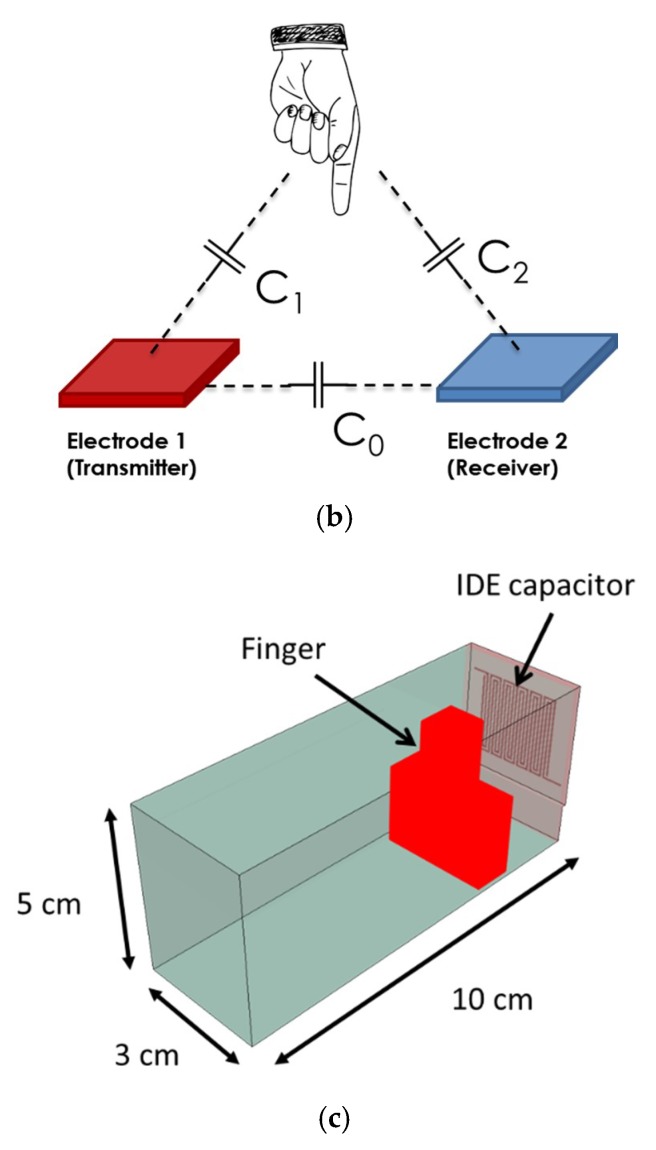
(**a**) Photo images showing the testing setup where a human finger is positioned at different positions above the IDE capacitor. (**b**) Illustration of the different capacitance paths as a human finger approaches the capacitor. C_0_ is the intrinsic capacitance and C_1_C_2_ are the parasitic capacitances. (**c**) FE model in the numerical simulation (finger and IDE capacitor indicated) with a size of 10 cm × 5 cm × 3 cm.

In this section, we describe the proximity sensing effect of the IDE capacitor through the disturbance of the E-field lines with objects placed at different distances from it. Figure 5a shows the testing setup photo images where the IDE capacitor was connected to a precision impedance analyzer (Agilent 4294A, Santa Clara, CA, USA) for capacitance measurement. The corresponding capacitance was measured as a human finger was moved between different distances from the surface of the IDE capacitor, as shown in Figure 5a. The proximity sensing mechanism is described as follows. Capacitive sensors use capacitive transducers to detect the proximity of a body, and are broadly classified in three modes: transmit mode, shunt mode, and loading mode [26]. Our setup behaved as a transmit mode where one side of the electrodes acted as a transmitter, and the other side as a receiver. Figure 5b shows an illustration of the different capacitance paths of the system as a human finger approached. The original capacitor electric field was now coupled to the receiver side through the human finger, creating two parasitic capacitances (C_1_ and C_2_) in series. Thus, the effective capacitance of the system increased based on the capacitance summation rule in a parallel configuration between the intrinsic capacitance C_0_ and the parasitic capacitances C_1_ and C_2_. C_0_, C_1_, and C_2_ were, in turn, dependent on the distance of the human finger from the capacitor due to the strength of the electric-field coupling. The further away the finger was from the capacitor, the weaker the coupling and the smaller the effective capacitance. In our experiment, a reduction of capacitance was seen as the finger moved towards the capacitor, while capacitance increased when the finger moved away from the capacitor. The proximity effect could be felt by the sensor from a distance as far as 28 cm. Compared with the dielectric property of air, human tissue is considered to be a conductive material. From the perspective of electrostatics, the boundary of human tissue was assumed to be a floating electrode, where the total electrical charge at the surface was equal to zero. The entire FE simulation model had a volumetric size of 10 cm × 5 cm × 3 cm, and the IDE capacitor, air, and the human finger model is shown in Figure 5c.

**Figure 6:**
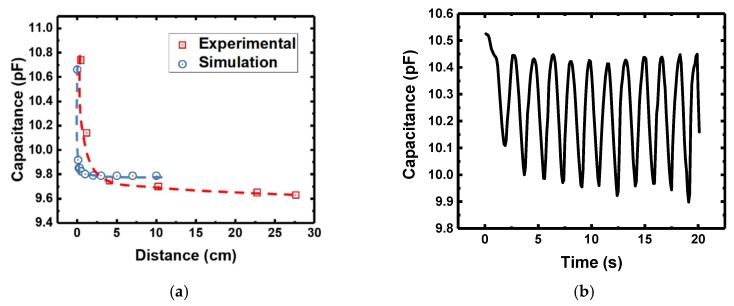
(**a**) Measured capacitance w.r.t. various distances a human finger was placed from the IDE capacitor, and (**b**) transient capacitance plot when a human finger hovered above the IDE capacitor in a cyclic manner at a height of ~1–3 cm above.

Figure 6a,b shows the capacitance versus distance of a human finger placed above the IDE capacitor up to 28 cm (both experimental and simulated indicated in legend), and time based capacitance measurements as the finger hovers above the IDE capacitor at a height of ~1–3 cm, respectively. In this simulation, a simple numerical finger model was constructed (Appendix). The main factors resulting in the discrepancy between the experimental and simulated values were due to the leaky fringe electrical field and the finger model difference. In addition, the simulated distance was limited at 10 cm due to calculation complexity beyond that. Nonetheless, the trend of the capacitance change agreed very well in both experimental and simulation, and the most sensitive region lay within 1 cm from the IDE capacitor. The disturbance in the field line resulted from the float electrical potential. When the distance between the finger model and sensor was within 1 cm, the leaky fringe electrical field around the finger became large, resulting in a significant capacitance change.

## 4. Demonstration of an IDE Capacitor Based Flexible Hybrid Respiratory System

Complementary Metal Oxide Semiconductor (CMOS) chips and printed circuit boards (PCBs) integrated with soft components allow them to be interfaced comfortably with the human body. The co-design of composite materials and electronic circuits/system in a monolithic form of a flexible hybrid electronic system provides the guideline for on-body wearables. In this section, we demonstrate a prototype hybrid flexible respiratory system by integrating the IDE capacitor to a rigid capacitance sensing circuit using external wires [29]. In subsequent measurements, the capacitance data measured by the chip is transmitted wirelessly via Wi-Fi protocol through a Raspberry Pi to a computer for readout. In the current setup, the IDE capacitor was connected to the external chip using tungsten wires. More reliable interconnects suitable for flexible system integration will be further investigated [30].

**Figure 7:**
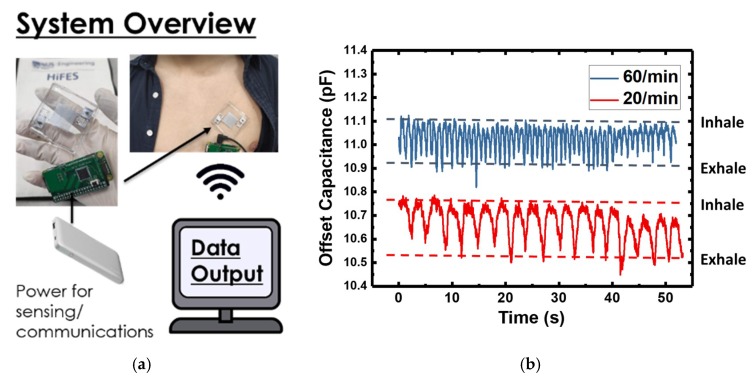
(**a**) System overview of the respiratory tracker with a photo image of the patch attached to a human chest, and (**b**) time dependence capacitance values measured by the capacitance chip as the human subject was breathing over 50 s. Two different respiratory rates were demonstrated as indicated in the legend.

The human body respiratory detection was achieved by utilizing the proximity sensing effect of the IDE capacitor, where the sensor was attached to the human chest. The expansion and contraction of the chest caused change in the effective dielectric constant of the surrounding medium and disturbed the electrical field lines. This allowed for the respiratory motion to be picked up in a straightforward manner. Hence, the sensor was placed at a location where chest motion was obvious in this demonstration. Figure 7a shows the photo image of the integrated system attached to a human chest, while Figure 7b shows the logged capacitance values by the circuitry over 50 s. During the respiratory rate tracking, the sensor and electronics were fully covered up by clothing. Notwithstanding the slight motion of the clothing during breathing, we did not observe any distortion to the acquired waveform. Although noise was present in the collected capacitance data, the undulating trace indicated the respiratory motion was clearly visible over a range of ~0.2 pF. We demonstrate two different respiratory rates of 20/min and 60/min as shown in Figure 7b. In both cases, the sensor responded adequately, and the captured rates matched with manual counting. The implementation shown here is straightforward but effective, thus paving the way for such sensor to be implemented in a wearable medical device.

## 5. Conclusions

In summary, we describe in detail the fabrication and electrical characterization of a soft PDMS-Galinstan based IDE capacitor. An FE simulation model was developed that allowed for dynamic boundary conditions to be applied, matching with experiments. With the Galinstan electrode completely encapsulated in the soft PDMS matrix, the IDE capacitor was mechanically robust to physical deformation as well as moisture. In addition to strain sensing in both the x and y axes, we show the proximity effect of such a class of soft sensors by modifying the surrounding dielectric medium. The disturbance of the electrical field lines by surrounding objects resulted in a change in effective dielectric constant, and consequently, a change in the capacitance. This effect allowed for spatial detection, and we showed experimentally that a sensing distance up to 28 cm can be achieved. The FE simulation elucidated the capacitance change mechanism and provide a guideline for more different applications. Finally, we demonstrate for the first time the use of an IDE capacitor based flexible hybrid electronics respiratory system utilizing the proximity effect. An accurate human breathing pattern was successfully tracked, paving the way for its use as a part of continuous health monitoring applications.

## Figures and Tables

**Figure A1:**
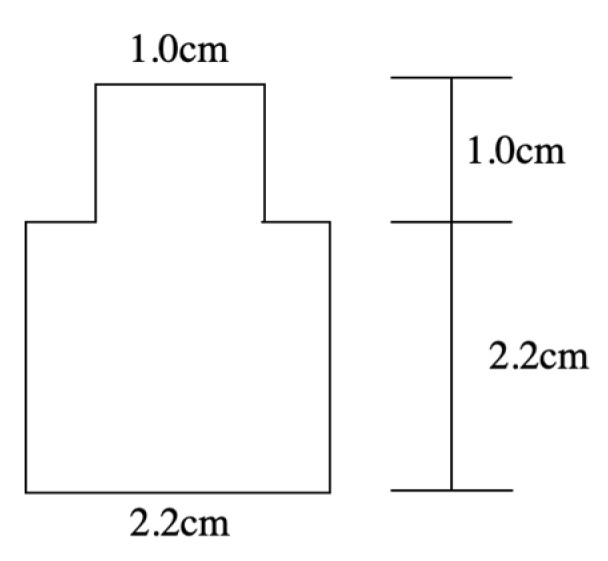
Shape and dimensions of the finger model. In this model, the thickness used was 1 cm.

## Appendix A

The finite element (FE) model was built up and simulated using commercial software Abaqus for non-linear FE analysis. We constructed two FE models to simulate the effect on the capacitance of the sensor due to deformation and proximity. In the FE model, the PDMS substrate was modelled using a C3D8RH element. PDMS was modelled using a hyperelastic material model (Mooney–Rivlin model in Abaqus) and was incompressible. The material parameters used were equivalent to the initial Young’s modulus = 1 MPa and Poisson’s ration = 0.49, obtained experimentally. Since the channels were completely filled with a nearly incompressible fluid, the surface-based fluid cavity capability was used, and the pressure applied by the fluid on the surface of channel was determined using the cavity volume.

To obtain the capacitance of the deformed sensor, the steady-state linear electrical–mechanical analysis (piezoelectric elements in Abaqus) was performed on the deformed solid mesh, where the piezoelectric constants were set to zero and the dielectric constant parameter was 2.62. In order to obtain the capacitance, an electrical potential ∆U was applied on the interfaces between the elastomeric matrix and liquid metal at the left and right channels. The charge Q in the channel was calculated using numerical simulation and the capacitance was then obtained as:

$$C=\frac{Q}{\Delta U}$$

In the proximity sensing simulation, the human finger was modelled as a simple model with dimensions as shown in Figure A1. In this model, the human finger was regarded as a near perfect conductor compared with the PDMS and air. To determine the capacitance of the position sensor, the human was set to a floating electrical potential, where the net surface electrical charge was zero. The resulting capacitance as a result of the finger distance to the sensor was extracted using the same methodology as the deformation model described in the previous paragraph.
